# 

**Published:** 1884-08

**Authors:** 


### INDEX.

### A.

PAGE.

Administration of Medicine, An unobjectionable Form for the.... 30
Arm Presentation in a Twin Case, Terminating by Spontaneous Expulsion .......................... 127
neurism of the Thoracic Aorta, with Death from Embolism from the Abdominal Aorta.......... 226
A Promise Generously Fulfilled... 524

### B.

Buffalo Medical and Surgical Association, Adjourned Meeting..... 15
Buffalo Medical and Surgical Association, Regular Meeting........ 61
Buffalo Medical and Surgical Association Regular Meeting........ 109
Berlin Letter.......................... 168
Buffalo Medical Library Association............................ 177
Barwell, Mr., on the Tubercular Degeneration of the Products of Strumous Synovitis............ 182
Buffalo Medical and Surgical Association, Regular Meeting....... 211
Buffalo Medical and Surgical Library Association.............. 234
Buffalo Medical and Surgical Association, Adjourned Meeting.... 254

PAGE.

Buffalo Medical and Surgical Association, Regular Meeting.........310, 361, 405, 452, 511, 555
Buffalo Obstetrical Society....379, 559
Berlin Letter..... .............. 263
Bursæ of Wrist, Removal of. C. C. F. Gay, M. D................. 550

### C.

Cholera and its Prevention ...... 41
Chloral Hydrate as a Vesicant.... 47
Cholera, Dr. Koch on ........... 66
Chronic Joint Disease............ 76
Campbell, F. R., A. M., M. D. The Influence of Seasons on Diseases in Buffalo............... 103
Chiseling of the Mastoid Process. 174
Cocaine, Muriate of.............. 184
Cæsarian Section, Stephen Y. Howell, M. A., M. D............... 193
Cocaine Hydrochlorate as Anæsthetic. A. A. Hubbell, M. D.. 241
Cæcum, Excision of.............. 569
Campbell, F. R., M. D. Sanitary Advantages of the Electric Light 151
Chloroform in Tic Douloureux.... 275
Commencing Hernia............. 276
Cholera and the Board of Health. 277
Campbell, F. R., M. D. Some Gynœcological Cases........... 307

PAGE.
Clinical Studies of the Early Stages of Inebriety. T. D. Crothers, M. D .......................... 445
Crothers, T. D., M. D. Clinical Studies of the Early Stages of Inebriety ...................... 445
Communication from Chas. Weil, M. D ........................ 464
Chemical versus Germ Theories of Disease. F. R. Campbell, A. M., M. D ........................ 483
Campbell, F. R., A. M., M. D. Chemical versus Germ Theories of Disease .................. 483
Cocaine in Vomiting of Pregnancy 519
Cocaine as an Antidote to Morphia .......................... 572

D.

Davidson, A. R., M. D. Hæmaturia as a Symptom of Diseases of the Genito Urinary Organs..... 1
Diphtheria. Specific Treatment of. 80
Diphtheria and Croup, Specific Treatment of.. ............... 81
Digitalis to prevent Excessive Desire for Copulation............. 83
Disinfection of Mail Matter....... 88
Death of Prof. Ed. Jæger......... 88
Davidson, A R. M. D. Rapid Dilatation of the Female Urethra for the Cure of Cystitis......... 105
Davidson, A. R., M. D., Clinical Lecture on Syhilides.............. 298
Dorland, E. T., M. D. Personal Experience with the Cholera Epidemic of 1852 53... .......... --
Dislocation of the Sternal End of the Clavicle, Upwards and Backwards........................ 173
Dietetic Treatment of the Nutritive Disorders in Children....... 229

PAGE.
Disappearance of Symptoms of Spinal Atrophy, following Removal of the Ovaries and Fallopion Tubes..................... 276
Diseases of the Scalp—The Syphilides. A. R. Davidson, M. D. 298
Diseases of the Testes. W. S. Tremaine, M. D.......... ...... 337
Dangerous Physicians............ 570

E.

Electrolytic Method, The Exact Value of the................ . 39
Editorial—Volume XXIV......... 40
Enlarged Kidney. Wm Pepper, M. D., LL. D.................. 57
Eulachon Oil.................... 280
Energy of Nerve and Brain. W. H. Pitt, A. M., M. D.......... 394
Erie County Medical Society, 410, 562

F.

Five Cases of Uterine Pregnancy Operated upon at the Time of pture.......................... 185
Foods, Concentrated............ 567

G.

Gallic Acid in Hemorrhage from the Urinary Organs............ 37
Gangrene Senile, Mr. John Hutchinson's Views on the Treatment of............................ 79
Gay, C. C F., M. D. Removal of Bursæ of the Wrist........... 550
Gangliar Disease of Joints........ 172
Gynecic Use of Hydrochlorate Cocaine ............................ 232
Gunshot Wounds of the Stomach—Successful Laparotomy......... 271

PAGE.

Grove, B. H., M. D. Some Experience with Cocaine Hydrochloride on the Eye................ 355
Girard, Alfred C., M. D., U. S. Army. Listerism and Another Step in Advance............... 385

H.

Hæmaturia as a Symptom of Diseases of the Genito Urinary Organs. A. R. Davidson, M. D... 1
Hubbell, A. A., M. D. Membranous and Diptheritic Conjunctivitis ........................ 9
Hospital with Circular Wards..... 39
Hutchinson's, Mr. John, Views on the Treatment of Senile Gangrene ........................ 79
Howell, Stephen Y., M. A., M. D. Cæsarian Section.............. 193
Hubbell, A. A., M. D. Cocaine Hydrochlorate Anæsthesia...... 241
Herpes Laryngis................. 270
Honors to a Buffalo Physician.... 283
Hoarseness and Loss of Voice as Symptoms. F. W. Hinkel, M. D. 435
Hinkel, F. W., M. D. Hoarseness and Loss of Voice as Symptoms. 435
High Temperature Preceding Abortion.......................... 518
Hydrangea, Lithiated............ 562

I.

Intestinal Diseases of Infancy. C. G. Stockton, M. D............ 541
Iodoform, A Dangerous Adulteration of...................... 84
Influence of Season on Diseases in Buffalo. F. R. Campbell, A. M., M. D........................ 103
Impaired Vesical Function....... 121

PAGE.

Illinois State Board of Health and Reform in Medical Education... 520

K.

Kilbourne, H. S., M. D., U. S. A. Two Cases of Cerebral Syphilis. 205
Koch's, Dr., Cultivation Experiments in Cholera Bacillus. Geo. W Lewis, Jr., A. B............ 343
Koch's Experiments for the Eradication of the Cholera Bacillus. Geo. W. Lewis, Jr., A. B...... 500

L.

Local Treatment of Laryngeal Tuberculosis .................... 124
Lesser Degrees of Chronic Pelvic Inflammations in Women...... 132
Lassing, H., M. D., Mosaic and Modern Dietary Laws...... ... 289
Lewis, Geo. W., Jr., A. B. Dr. Koch's Cultivation Experiments in Cholera Bacillus............ 343
Listerism and Another Step in Advance. Dr. Alfred C. Girard, U. S. Army.............................
Legal Control of Medical Practice by a State Examination ....... 425
Longevity in the Different States.. 472
Lewis, Geo. W., Jr. Koch's Experiments for the Eradication of the Cholera Bacillus........... 500
Levis, R. J., M. D. Metallic Splint......................... 564

M.

Membranous and Diptheritic Conjunctivitis. A. R. Hubbell, M. D. 9
Medical Colleges............... 133
Medical Education. Wm S. Tremaine, M. D.................. 145
Medical Science in China.... .... 180

PAGE.
Medical Society of the County of New York.................... 282
Mosaic and Modern Dietary Laws. H. Lossing, M. D.............. 289
Macniel, Dugald, M. D. In Memoriam.......................... 430

### N.

New Method of Removing Nasal Polypi......................... 82
Niagara Medical College... ..... 87
Notes on Trachelorrhaphy........ 222
Niagara Medical College.......... 335
New Orleans Meeting of the American Medical Association....... 377

### O.

Overdose of Chloral during Parturition .......................... 84
On the Cause and Curability of Locomotor Ataxia....... ..... 127
Ovariotomy with Extirpation of one Kidney ...................... 133

### P.

Pelvic Abscess in the Female, W. W. Potter, M. D............. 531
Pepper, Wm., M. D., LL. D. Enlarged Kidney................. 57
Pepper, Wm., M. D., LL. D. Pneumonia—A Clinical Lecture. 97
Personal Experience with the Cholera Epidemic of 1852-3. E. T. Dorland, M. D................ 155
Placenta Prævia................. 174
Puerperal Eclampsia, Treated by Veratrum Viride................ 185
Proceedings of the Erie County Medical Society, Annual Meeting 321
Pathology and Treatment of Extra-Uterine Pregnancy...... .. ... 470

PAGE.
Pneumonia, Clinical Lecture. Wm. Pepper, M. D .... ...... ..... 97

### R.

Radical Treatment of Hernia by Excision of the Sac............ 74
Rabies Inocculation............. 77
Rapid Dilatation of the Female Urethra for the cure of Cystitis. A. R. Davidson, M. D......... 105
Repair of Wounds and Fractures in Aged Persons.................. 126
Reports of Contagious Diseases, to the Board of Health........... 333
Ricord, M., on Cholera........... 379
Rapid Dilatation of the Uterine Canal for Dysmenorrhœa and Sterility...................... 467

### S.

Stockton, C. G., M. D. Treatment of the Intestinal Diseases of Infancy ------------------------- 541
Supra Pubic Lithotomy. W. S. Tremaine, M. D............... 49
Specific Treatment of Diphtheria. 80
Specific Treatment of Diphtheria and Croup...................... 81
Surgical and Orthopœdic Treatment of Infantile Paralysis..... 170
Syllabus of the Treatment of Fractures of the Cranium........... 186
Sanitary Advantages of the Electric Light. F. R. Campbell, M. D... 251
Some Gynœcological Cases. F. R. Campbell, M. D................ 307
Strangulated Umbilical Hernia... 331
Some Experience with Cocaine Hydrochloride on the Eye. B. H. Grove, M. D.................. 355
Shall Midwives be Licensed....... 423
Spontaneous Rupture of the Membranes at Full Term............ 519

### T.

PAGE.

Tremaine, W. S., M. D., on Fracture of the Skull.................. 553
Treatment of Chancroid and Syphilis...... ................. 20
Tremaine, W. S., M. D. Supra Public Lithotomy......... .... 46
Treatment of Lupus ............. 83
Transactions of the Buffalo Obstetrical Society................... 116
Tremaine, Wm. S., M. D Medical Education..................... 145
Transactions of the Buffalo Obstetrical Society............ .... 164
Two Cases of Cerebral Syphilis. H. S. Kilbourne, M.D., U. S. A. 205
Transactions of the Buffalo Obstetrical Society... ................ 217
Temperature of the Extern l Auditory Canal in Disease.......... 228
Tubal Pregnancy, A Case of...... 23c
Tubercular Origin of Joint Diseases ...................... ... 230

PAGE.

Transactions of the Buffalo Obstetrical Society..................
.......259, 312, 367, 414, 458, 513
Treatment of Whooping Cough... 520
Treatment of Cancer of the Rectum...................... 274
Tremaine, W. S , M. D. Diseases of the Testes.................. 337
To the Meeting of the American Medical Association............ 428

### U.

Uræmic Amaurosis.............. 85
Uterus, Removal of.............. 568

### W.

Washington Obstetrical and Gynecological Society.... .......... 234
Weight as an Indication of the Character of the Risk for Life Insurance..................... 473

